# Improving healthcare sustainability using advanced brain simulations using a multi-modal deep learning strategy with VGG19 and bidirectional LSTM

**DOI:** 10.3389/fmed.2025.1574428

**Published:** 2025-04-10

**Authors:** Saravanan Chandrasekaran, S. Aarathi, Abdulmajeed Alqhatani, Surbhi Bhatia Khan, Mohammad Tabrez Quasim, Shakila Basheer

**Affiliations:** ^1^Department of Computer Science and Engineering, SRM Institute of Science and Technology, Ramapuram, Chennai, India; ^2^Department of Computer Science and Engineering (Data Science), Dayananda Sagar College of Engineering, Bangalore, India; ^3^Department of Information Systems, College of Computer Science and Information Systems, Najran University, Najran, Saudi Arabia; ^4^School of Science, Engineering, and Environment, University of Salford, Salford, United Kingdom; ^5^University Centre for Research and Development, Chandigarh University, Mohali, India; ^6^Centre for Research Impact and Outcome, Chitkara University Institute of Engineering and Technology, Chitkara University, Rajpura, India; ^7^Department of Computer Science and Artificial Intelligence, College of Computing and Information Technology, University of Bisha, Bisha, Saudi Arabia; ^8^Department of Information Systems, College of Computer and Information Science, Princess Nourah bint Abdulrahman University, Riyadh, Saudi Arabia

**Keywords:** brain tumor classification, multi-modal learning, VGG19, bidirectional LSTM, LightGBM, MRI imaging, deep learning, ensemble learning

## Abstract

**Background:**

Brain tumor categorization on MRI is a challenging but crucial task in medical imaging, requiring high resilience and accuracy for effective diagnostic applications. This study describe a unique multimodal scheme combining the capabilities of deep learning with ensemble learning approaches to overcome these issues.

**Methods:**

The system integrates three new modalities, spatial feature extraction using a pre-trained VGG19 network, sequential dependency learning using a Bidirectional LSTM, and classification efficiency through a LightGBM classifier.

**Results:**

The combination of both methods leverages the complementary strengths of convolutional neural networks and recurrent neural networks, thus enabling the model to achieve state-of-the-art performance scores. The outcomes confirm the efficacy of this multimodal approach, which achieves a total accuracy of 97%, an F1-score of 0.97, and a ROC AUC score of 0.997.

**Conclusion:**

With synergistic harnessing of spatial and sequential features, the model enhances classification rates and effectively deals with high-dimensional data, compared to traditional single-modal methods. The scalable methodology has the possibility of greatly augmenting brain tumor diagnosis and planning of treatment in medical imaging studies.

## Graphical abstract

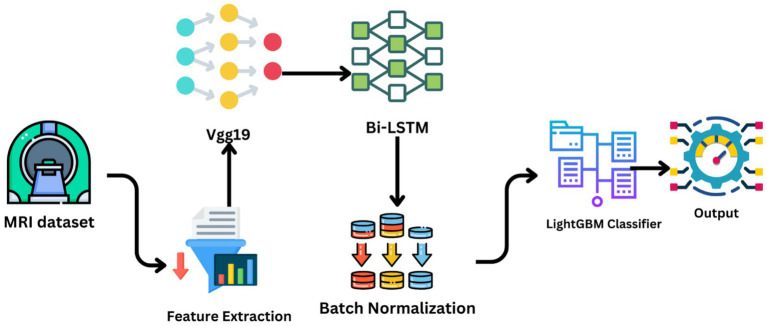

## 1 Introduction

**Figure 1:**
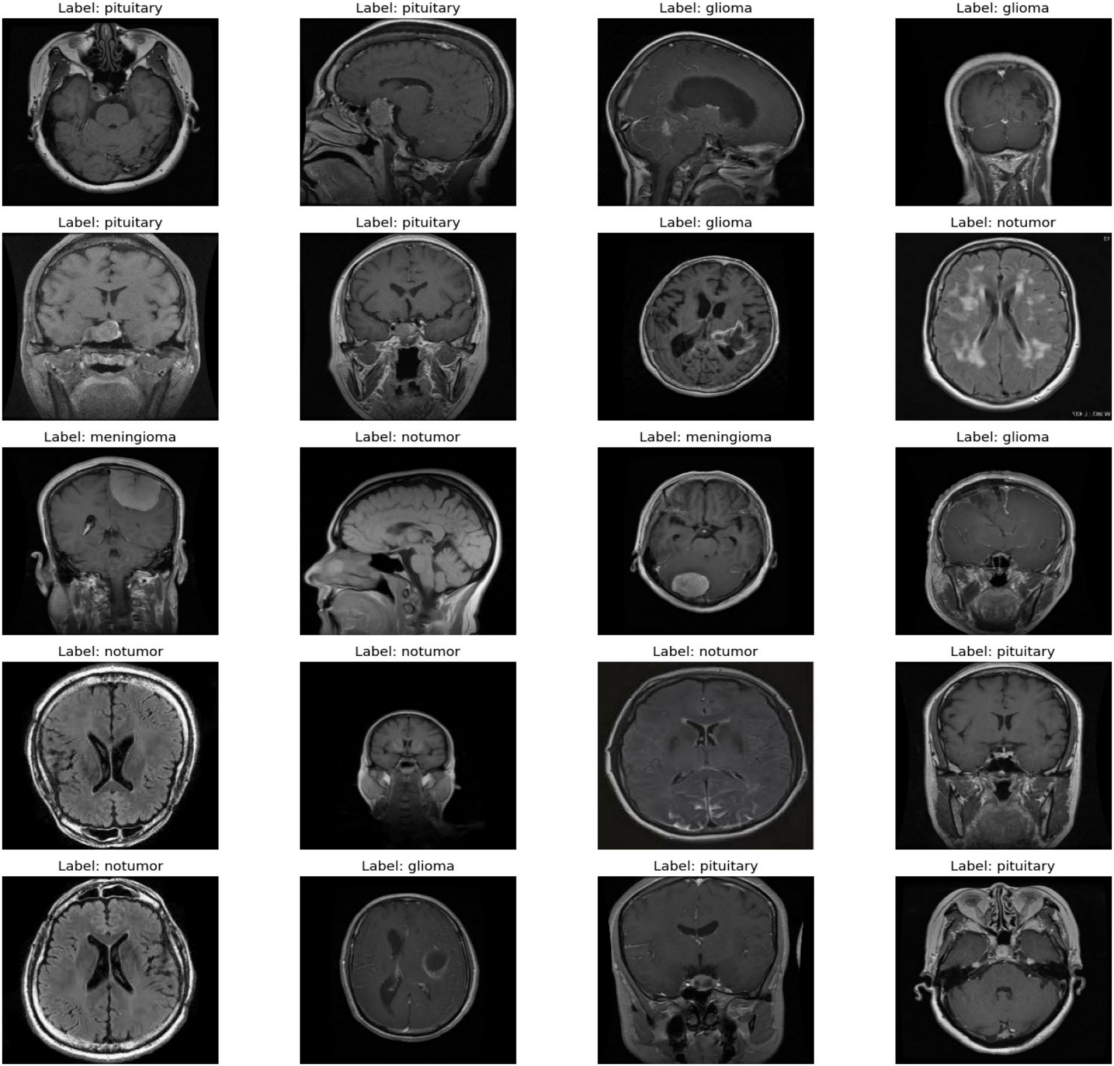
A sample of images from the dataset.

Brain tumor segmentation from MRI images is an important component of medical imaging, and serious consequences follow for the diagnosis, treatment, and prognosis of the patient. The heterogeneity and complexity of brain tumors and the high-dimensionality of MRI data pose significant challenges to traditional diagnostic approaches. These include problems like tumor variability in appearance due to size, shape, and location, which can complicate detection and classification. Diagnosis with a human expert is generally cumbersome, subjective, and prone to error, and traditional machine learning approaches rely on manually designed features, which are prone to missing out on the complexities of MRI data. It uses advanced preprocessing techniques like image normalization and data augmentation to enhance training and model stability. Improvements in machine learning and deep learning enabled the automation and accurate classification of brain cancers. In this work, a new multi-modal approach is introduced that uses deep learning and ensemble learning methods to tackle these challenges, thus providing a scalable and effective approach to classifying brain tumors (1). Employing bidirectional long-term memory networks to represent sequential dependencies in MRI slices, deep convolutional neural networks to enhance spatial feature extraction, and LightGBM for high-dimensional data classification in an efficient way, the proposed VGG19-BiLSTM-LightGBM model. This multimodal approach synergistically improves brain tumor categorization by combining the strengths of each model component, thereby enhancing the model’s ability to handle the intricacies of MRI data and improving diagnostic accuracy. Figure 1 shows the brain tumor images from the dataset.

The motivation for this work is the limitation imposed by existing techniques due to their inability to transcend such limitations. Because traditional diagnostic techniques, though effective within their confines, suffer from the heterogeneity of tumor size, shape, and location (2), and single-modal techniques account only for spatial or sequential characteristics and cannot harness the full richness of MRI image information, therefore, a method has to be developed those accounts for the interplay between spatial and sequential factors. This is capable of building more robust and precise classification by including these techniques as a multi-modal technique. Ensemble learning algorithms like LightGBM provides stable classification, effectively handling the high-dimensional data and aggregating the strengths of individual models (3).

This work centralizes to the creation of a multi-modal deep learning architecture for brain tumor classification that synergistically integrates the spatial and sequential features of MRI images. Spatial feature extraction was carried out through a pre-trained VGG19 model, thereby making it feasible and accurate for representing MRI images. To improve the model’s capacity to learn the underlying patterns, a bidirectional LSTM layer is used to monitor temporal relationships among the extracted features (4).

This work is on the integration of multiple modalities, such as sequential modeling using Bidirectional LSTM and spatial feature learning using VGG19. The drawbacks of the traditional methods are alleviated through this work by giving an end-to-end solution to brain tumor classification. MRI image description becomes more realistic with the use of an integration of multiple modalities. The classification performance is further augmented by LightGBM being utilized as a final classifier to enable effective processing of high-dimensional data (5). Large and high-dimensional data can be handled using the proposed framework, which renders it easy to implement on actual healthcare challenges. High validation accuracy with minimal amounts of loss indicates its generalization capabilities to unseen data. The following sections of this paper are classified as given below. Section 2 gives an overview of the major research on brain tumor classification including deep learning and ensemble learning techniques. Section 3 provides a thorough explanation of the suggested methodology, i.e., data preparation, feature extraction, and classification. Section 4 discusses the experimental results, including performance metrics and comparisons with baseline models.

## 2 Literature review

The field of brain tumor classification from MRI scans has experienced tremendous expansion in the recent past with momentum building for the application of deep learning and machine learning techniques. Traditional methods in brain tumor diagnosis have employed close to all visual inspection by radiologists, not just time-consuming but also prone to human error (6). Such methods tend to employ extraction of inherent features like texture, shape, and intensity that might not reflect the complex patterns present in medical images. Therefore, there has been a move toward automated methods that take advantage of the strengths of deep learning to achieve improved accuracy and efficiency.

Convolutional Neural Networks have become a backbone of modern medical image analysis. Their ability to learn spatial features automatically from images has made them particularly suitable to applications such as tumor detection and classification (7). From pre-trained CNN models, VGG19, ResNet, and Inception can be broadly applied in medical imaging because they can generalize toward a large range of datasets. The early layers typically freeze, and the final layers are fine-tuned on the target dataset toward a specific application, such as the classification of brain tumors. This reduces not only the computational cost but also enhances performance with knowledge gained from large-scale datasets, such as ImageNet.

While CNNs excel at capturing spatial features, they may not fully exploit the sequential or temporal dependencies present in medical images. LSTMs are designed to represent sequential data, making them optimal for identifying temporal trends in medical images (8). Bidirectional LSTMs, which process data in both forward and backward directions, have been shown to further enhance performance by capturing more comprehensive dependencies. The combination of CNNs and LSTMs has been explored in various medical imaging tasks, including brain tumor classification, where it has demonstrated superior performance compared to standalone models. Table 1 shows the exiting studies through multiple techniques.

**Table 1:** Existing studies from different techniques.

Study	Objective	Remark
Maqsood et al. (13)	To present an automated technique for precise brain tumor identification and classification by using deep learning and MRI.	The method achieved high accuracy (97.47 and 98.92%) and outperformed prior methods.
Jiang et al. (14)	To develop SwinBTS, a 3D medical image segmentation approach combining transformers and CNNs for brain tumor classification.	SwinBTS beat state-of-the-art algorithms on BraTS 2019, 2020, and 2021 datasets.
Zhu et al. (15)	Present a brain tumor segmentation approach that integrates deep semantics and edge information in multimodal MRI.	The method outperformed state-of-the-art methods on BraTS benchmarks.
Zhang et al. (16)	Introducing mmFormer: A Transformer-based approach to strong multimodal brain tumor segmentation with incomplete modalities.	mmFormer outperformed state-of-the-art approaches, particularly with missing modalities.
Razzaghi et al. (17)	A multimodal deep transfer learning system that can be used with MRI brain image processing should have domain flexibility.	The strategy outperformed equivalent algorithms on IBSR and Figshare datasets.
Ali et al. (18)	Analyze the progresses in brain tumor segmentation, feature extraction, and classification using MRI along with deep learning.	Highlights the move from traditional approaches to deep learning and hybrid methodologies.
Peng and Sun (19)	To propose AD-Net, an autonomous weighted dilated convolutional network for multimodal brain tumor feature extraction.	Achieved high Dice scores (0.90, 0.80, 0.76) on BraTS20 dataset.
Fang and Wang (20)	To propose MFF-DNet, a dual-path network for multi-modal feature fusion in brain tumor segmentation.	Achieved high precision (0.92 and 0.90) for whole tumor and core tumor segmentation.
Hossain et al. (21)	To propose a strategy for brain tumor segmentation using 3D U-Net and ResNet50 with image fusion.	Achieved high accuracy (98.96% for ResNet50, 97.99% for 3D U-Net).
Liu et al. (22)	To present SF-Net, a multi-task model for brain tumor segmentation leveraging segmentation-fusion.	Achieved higher segmentation accuracy than VAE-based approaches on BraTS 2020.
Prasad et al. (23)	To enhance medical imaging capabilities using a CNN-based approach for detecting and classifying brain tumors.	The proposed model achieves superior accuracy, recall, F1-score, and precision compared to traditional methods, contributing to more effective brain tumor analysis.
Kargar Nigjeh et al. (24)	To optimize brain tumor classification using deep learning models and advanced image enhancement techniques.	The study demonstrates high classification accuracy (95%) and provides insights into the strengths and limitations of various deep learning architectures for medical imaging.
Sharma et al. (25)	To improve efficiency in brain tumor categorization through a hybrid model approach.	The model achieves 97% classification accuracy by integrating multiple learning techniques, enhancing robustness in tumor classification.
Bibi et al. (26)	To address computational inefficiencies and improve classification accuracy through a transfer learning approach.	The InceptionV4 model achieves 98.7% accuracy, significantly improving diagnostic precision and reducing computation time.
Albalawi et al. (27)	To develop an advanced CNN architecture for more accurate and efficient brain tumor diagnosis.	The CNN model achieves an exceptional 99% accuracy, marking a major advancement in automated MRI analysis and early tumor detection.

Ensemble learning methods have also found relevance in medical image analysis because they can enhance classification accuracy and robustness. Techniques such as Random Forests, Gradient Boosting, and LightGBM combine the predictions of many models to produce more accurate and reliable results. LightGBM is specifically widely used because of its ability to work on enormous datasets and high-dimensional data (9). By combining deep learning models with ensemble techniques, scientists have been able to develop hybrid frameworks that leverage the strengths of both methods.

While great advances have been made, brain tumor categorization still presents some challenges. One of the most significant is that the tumors are very variable in how they look, which could vary greatly by size, shape, and even placement. All this variability makes it difficult to build a model that generalizes well over all datasets. Because the dimension of the MRI data is high, their computation presents serious challenges in particular when a lot of them is involved. Methods such as flipping, rotating and adjusting the brightness randomly, used to enlarge training data variety while preventing overfitting have commonly been employed for overcoming this difficulty (10). The third is interpretability in medical imaging models.

Multi-modal combination is a key component in improving categorization. Multi-modal techniques provide a more comprehensive explanation of the underlying issue by combining multiple data modalities, such as MRI images, clinical data, and genomic data. Multi-modal techniques have been shown to perform better than single-modal approaches in the categorization of brain tumors by complementarily gathering information from diverse data sources. It is presently known that the fusion of MRI images with clinical data, like the patient’s age and medical history, improves classification performance and provides more individualized predictions (11). Brain tumor classification has greatly improved in the past few years due to advances in deep learning, ensemble learning, and multi-modal methods.

## 3 Methodology

**Figure 2:**
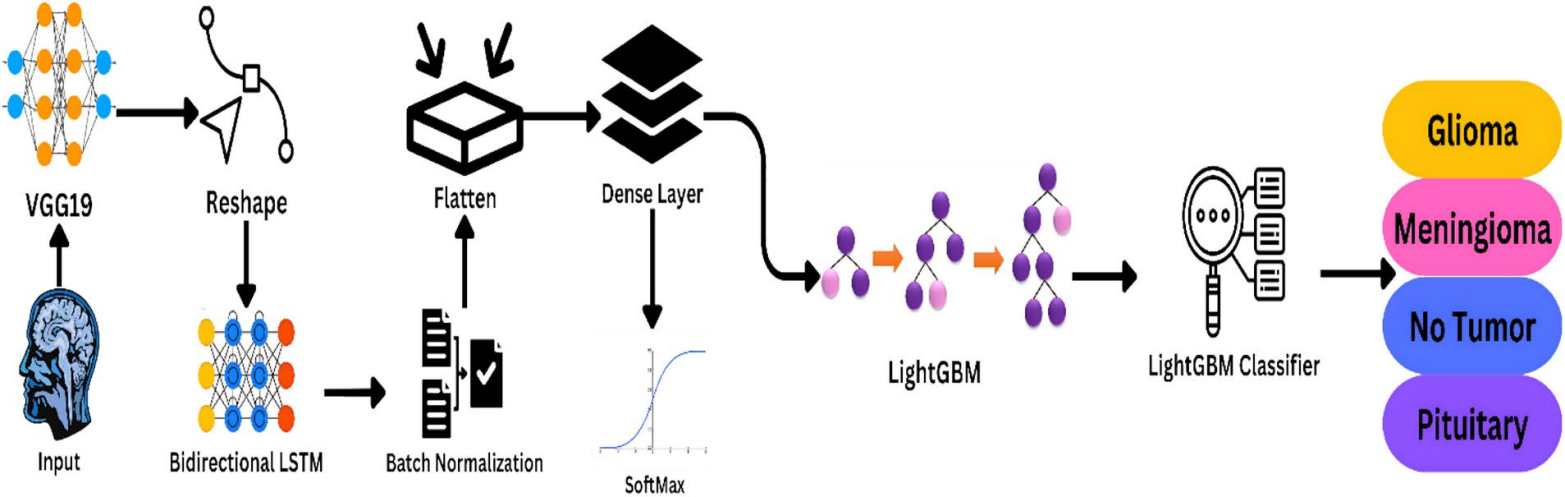
Workflow of the proposed model the framework is ideal for real clinical applications as it strives for high accuracy and generality.

The multi-modal nature of the proposed method for MRI-based brain cancer diagnosis is becoming increasingly popular. For sequence modeling and feature extraction, it uses deep learning models like VGG19 and Bidirectional LSTM, for classification, it uses LightGBM. Figure 2 illustrates a step-by-step overview of the preferred model’s approach.

### 3.1 Dataset description

The 7,023 MRI images of the human brain that make up the Brain Tumor MRI dataset are split into four categories: pituitary, meningioma, glioma, and no tumor. Glioma tumors are made up of glial cells, while Meningioma malignancies arise from the meninges, protective coverings of the brain and spinal cord. The Pituitary class contains cancers that originate in the pituitary gland, a small gland at the base of the brain that is responsible for the production of hormones. The “No Tumor” class contains normal brain scans to act as a control set for comparative analysis. The data were intentionally divided into training, validation, and testing sets in 70, 15, and 15% ratios, respectively. The ratio of splitting was aimed at achieving a trade-off between enough training data to learn the model parameters well and adequate validation and test data to analyse the performance and generalizability of the model comprehensively. The significant portion dedicated to training ensures deep learning models, which demand huge amounts of data, get well-trained. Equal partitioning of the rest of the data for validation and testing helps refine model parameters and test the model on data not seen by it, reducing the risk of overfitting. The method also ensures that the evaluation measures capture the model’s ability to function under varying conditions, thus offering a truer measure of its potential effectiveness in actual use. The wide scope of categorization ensures total research over a wide range of common situations of the brain, thus enhancing representativeness when the model is used in practical applications. The dataset, though, has its limitations in the shape of potential class imbalance and heterogeneity in tumor locations and sizes, which could hinder learning as well as predictive capacity. Three primary sources make up this dataset: the SARTAJ dataset, which initially consisted of glioma images but contained inconsistencies that led to their replacement with images sourced from figshare; the Br35H dataset, which provides images for the “No Tumor” class; and figshare, which offers images for glioma, meningioma, and pituitary tumors. It is thought that this data would make it possible to design automated systems for the classification of brain cancers with proper early detection and a proper diagnosis. It has been divided into training and test sets, with images resized to 224 × 224 pixels for deep learning models such as VGG19. The dataset’s size and heterogeneity render it a valuable source of information upon which researchers and medical imaging professionals can formulate generalizable and robust brain tumor classification algorithms.

### 3.2 Data preprocessing

The first preprocessing operation is scaling of images. The MRI images in the dataset are resized to a uniform size of 224 × 224 pixels. Standardization is necessary because deep learning models like VGG19 need to have fixed input sizes. Resizing enables all images to be compatible with the model architecture, thus enabling effective batch processing during training. Resizing also reduces the computational complexity by downsampling high-resolution images without significantly reducing their quality. Equation 1 shows the resizing of images.


(1) $$I^{\prime }=\mathrm{resize}\left(I,h,w\right)$$

The resized images are then normalized, which is the process of scaling pixel values to a particular range. In this case, pixel values are normalized to the range [0, 1] by dividing the pixel intensity by 255. Normalization is necessary since it ensures the input data have a fixed scale, which improves the convergence of the model while training. If not normalized, the model will fail to learn since the magnitudes of pixel values vary from image to image. Equation 2 illustrates the formula to normalize the images.


(2) $$x^{\prime }=\frac{x-\min\left(x\right)}{\max\left(x\right)-\min\left(x\right)}$$

In order to improve the strength and variety of the dataset, data augmentation techniques are applied. Data augmentation is artificially conducted to enlarge the size of the training dataset by creating multiple copies of the original images. This process not only addresses the issue of limited data in medical imaging but also simulates varying imaging conditions, which helps in building a robust model. The data augmentation techniques applied in this system not just random horizontal and vertical flips, but also random horizontal and vertical flip, which mimic different brain orientations; random change in brightness, which introduces lighting variability; random change in contrast, which changes the difference in intensity of pixels; random change in saturation, which changes the colour intensity; and random change in hue, which changes the tonal quality of images. These transformations are essential for training the model to recognize tumors under different imaging conditions and enhance its ability to generalize across new, unseen datasets. Equation 3 represent the mean and standard deviation of the pixel values in the image. Equation 4 applies a flip transformation along a specified axis (horizontal or vertical) to the image. Equation 5 brightens the image by adding a constant β , being possibly positive (to brighten) or negative (to darken). Equation 6 adjusts the pixel values of $I^{\prime \prime \prime }$ to change the contrast.


(3) $$I^{\prime \prime \prime \prime }=I^{\prime \prime \prime }\cdot R\left(\theta \right)$$

(4) $$I^{\prime \prime \prime \prime \prime }=\mathrm{flip}\left(I^{\prime \prime \prime }\mathrm{,axis}\right)$$

(5) $$I^{\prime \prime \prime \prime \prime \prime }=I^{\prime \prime \prime }+\beta$$

(6) $$I^{\prime \prime \prime \prime \prime \prime \prime }=\alpha \cdot \left(I^{\prime \prime \prime }-\mu \right)+\mu$$

Data preprocessing pipeline is built to transform raw MRI images to an appropriate form for deep learning models. By resizing, normalizing, augmenting, and organizing the data, the pipeline enables the model to learn and generalize effectively to unseen new data. These preprocessing steps are important to achieve high accuracy and robustness in brain tumor classification and are therefore an integral part of the proposed approach.

### 3.3 Model architecture

**Figure 3:**
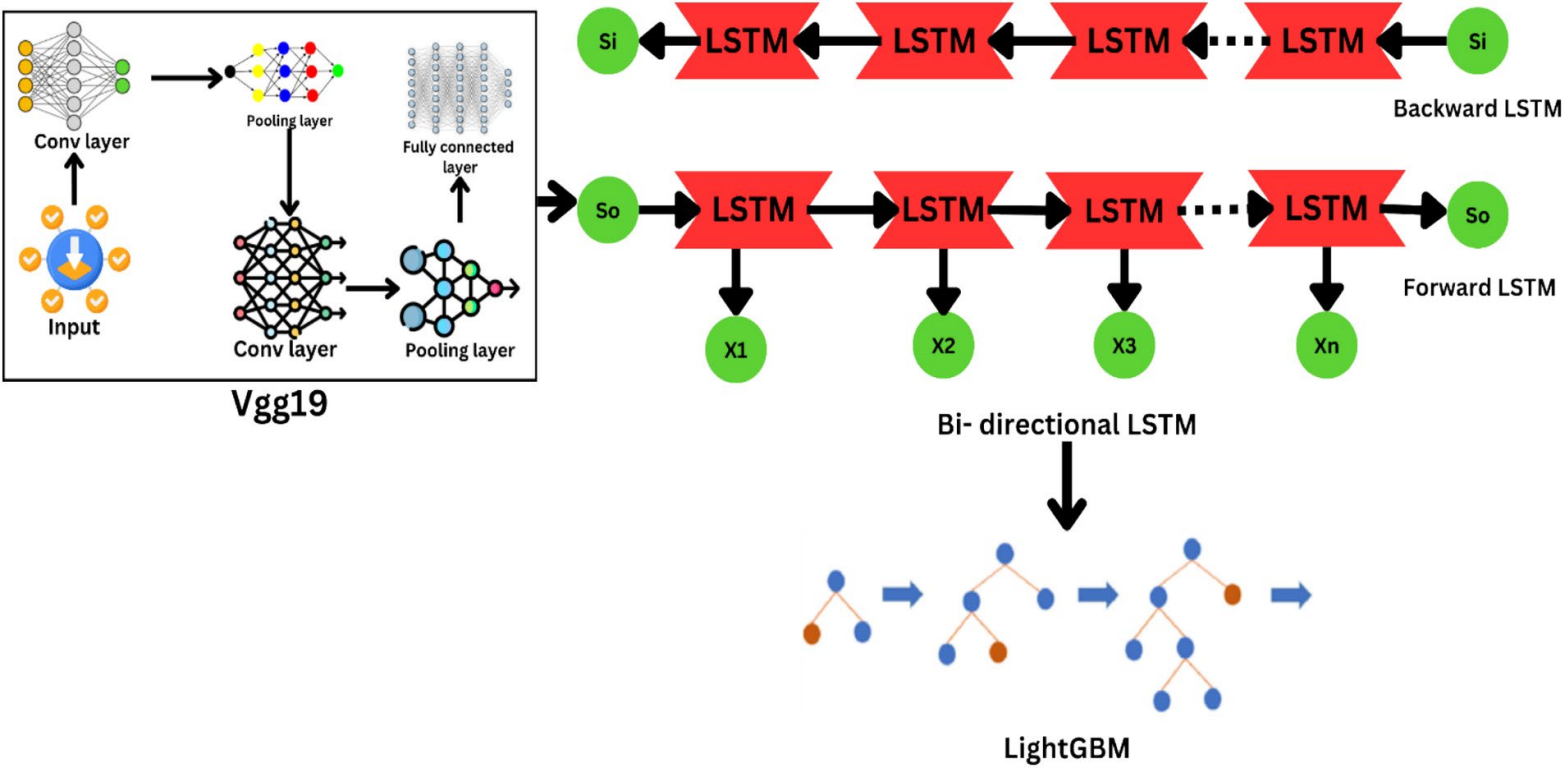
Architecture of the VGG19-BiLSTM-LightGBM framework.

The suggested classification system of brain tumors uses a combination of deep learning models to achieve great accuracy and robustness. The construction of automated approaches for the classification of brain cancers with sufficient early detection and precise diagnosis is anticipated to be enabled by dataset. For deep learning models such as VGG19, it has been split into training and test sets, and the photographs have been resized to 224 × 224 pixels. Due to the volume and diversity of the dataset, researchers and medical image professionals can utilize it to construct valid and generalisable analysis. Figure 3 shows the Model Architecture of VGG19-BiLSTM-LightGBM Framework.

The technique of converting raw MRI scans into an applicable set of features suitable for classification is referred to as feature extraction, and it is the initial step of the deep learning pipeline. A pre-trained VGG19 model is used to do this. VGG19 is a very deep convolutional neural network (CNN) architecture that has been widely applied in computer vision tasks due to its capability to extract hierarchical features from images. The architecture of VGG19 comprises 19 layers, including 16 layers of convolutional layers, 3 of fully connected layers, and 5 max-pooling layers. On this model, they apply pre-training from ImageNet dataset, incorporating over 1 million images in 1,000 categories. The pre-trained model gives the feature of identifying general features, such as edges, textures, and shapes, which can be further fine-tuned for any other task. In this case, it is for medical image analysis. Equation 7 gives the output size of a convolutional layer.


(7) $$O=\frac{W-K+2P}{S}+1$$

Transfer learning is employed in the suggested framework to fine-tune the VGG19 model for brain tumor classification. Transfer learning is the reuse of a pre-trained model with fine-tuning for a task. The model is set up to receive input images of size 224 × 224 pixels. The pre-trained weights are imported, to focus on extracting the most relevant features for brain tumor classification, only the early convolutional layers of the model are frozen, allowing the deeper layers, which are more specific to the task at hand, to adjust during the training process. This keeps the model to retain the common features learned from ImageNet while learning task-specific features in the later layers. The VGG19 model processes the input MRI images and extracts high-level spatial features from its final convolutional layer. These features represent the most discriminative aspects of the images, such as tumor boundaries, texture, and intensity variations. The output of the VGG19 model is a feature map with dimensions 7 × 7 × 512, which is then passed to the next stage of the pipeline for further processing. To effectively use both sequential and spatial information, a Bidirectional LSTM layer has been added within the pipeline. LSTMs are a family of RNNs, the architecture of which is well-suited to the modeling of sequence data. Adding a Bidirectional LSTM allows the model to not only extract forward temporal dynamics but also backward dynamics, giving complete insight into sequence data. To enhance the ability of the model to learn the inherent patterns, a bidirectional LSTM layer is employed to track temporal relationships between the extracted features (4). This project is on the fusion of various modalities, like sequential modeling by Bidirectional LSTM and spatial feature learning by VGG19. The shortcomings of the conventional methods are overcome through this project with the provision of an end-to-end solution to brain tumor classification. MRI image description is made more realistic with the provision of an integration of various modalities. The classification efficiency is also enhanced through the use of LightGBM as a final classifier for efficient handling of high-dimensional data (5) using Equation 8. High-dimensional and large data are handled using the proposed framework, making it simple to deploy on real-life healthcare problems.


(8) $$\hat{x_{i}}=\gamma \left(\frac{x_{i}-\mu_{B}}{\sqrt{\sigma_{B}^{2}+\epsilon }}\right)+\beta$$

The last layer of classification takes the flattened output of the LSTM layer in the form of a 1D vector. It does this so that the features are brought in a form that allows easy classification. It is also designed for progressive learning. It allows the model to incrementally update its knowledge base whenever there is new information without rigid retraining needs. The inclusion of the LightGBM classifier within the model is highly significant in this case, as this classifier supports online learning environments. This aspect allows the model to update continuously with new data, hence enhancing its prediction with the passage of time. This is a highly significant feature in medical imaging, where shifting patterns of data require flexible models that can update with minimal downtime and computational costs.

To find the brain tumors consistently, the features that are extracted are used to train a LightGBM classifier, which is the final step in the deep learning process. A very good gradient boosting library capable of handling large high-dimensional data is known as LightGBM. The trained VGG19 and LSTM layers are used for building another feature extraction model. The gradient descent update rule is found in Equation 9. The logistic loss function for binary classification is found in Equation 10.


(9) $$\theta :=\theta -\eta \cdot \nabla_{\theta }J\left(\theta \right)$$

(10) $$l\left(\hat{y},y\right)=\sum_{i=1}^{n}\left[y_{i}\log\left(1+e^{-\hat{y_{i}}}\right)+\left(1-y_{i}\right)\log\left(1+e^{\hat{y_{i}}}\right)\right]$$

The features extracted are standardized with StandardScaler, thus obtaining a zero mean and unit variance for all the variables. This step is essential for maximizing the LightGBM classifier’s performance since it ensures that each feature contributes evenly to the classification process. With default hyperparameters, i.e., 200 estimators and a learning rate of 0.05, the LightGBM classifier is trained on scaled features. The retrieved features are used to train the algorithm to categorize different types of tumors. LightGBM is employed because it can generate precise and reliable predictions and is effective at managing big datasets. The operational flow and interdependencies between the various components of this multi-modal deep learning technique for MRI-based brain tumor classification are outlined sequentially in Algorithm 1.

**Algorithm 1:**
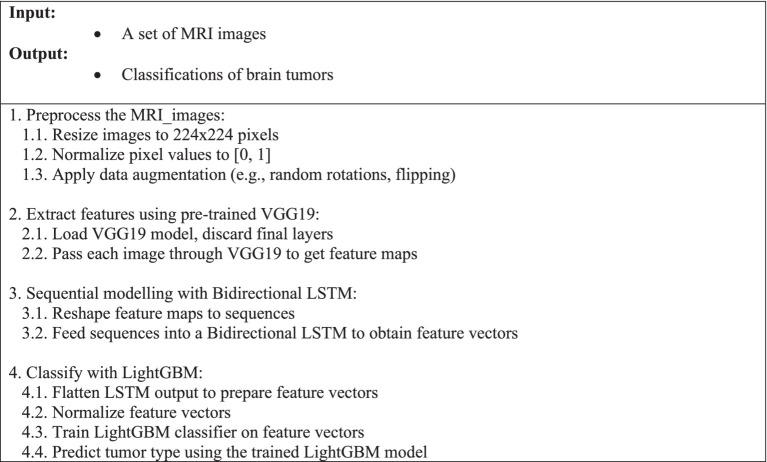
Multi-modal deep learning method for classifying brain tumors based on MRI.

The training process of the proposed VGG19-BiLSTM-LightGBM framework involves a multi-stage pipeline designed to optimize the model’s performance and generalization capabilities. This uses pre-trained VGG19 as the spatial feature extractor from the MRI images with all layers frozen so that weights learned during ImageNet can be preserved. Features from these layers are passed to the Bidirectional LSTM layer, which then encodes the temporal dependencies, followed by repeated processes of Batch Normalization and Flattening so that the data is made ready for classification. Equations 11, 12 can be used to compute the accuracy and precision of the model, respectively, which are two key parameters that can establish the efficiency of the model for real-world implementation.


(11) $$\mathrm{Accuracy}=\frac{TP+TN}{TP+TN+FP+FN}$$

(12) $$\mathrm{Precision}=\frac{TP}{TP+FP}$$

The whole pipeline is trained over the Brain Tumor MRI Dataset. To enhance training data variations, the entire dataset has methods applied that consist of random flips in any two planes and various combinations of changing brightness and contrast. Equations 13, 14 compute recall and F1-score thus yielding more criteria that are essential in judging performance concerning the positive values correctly discovered but at some expense in recall/precision ratio (12).


(13) $$\mathrm{Recall}=\frac{TP}{TP+FN}$$

(14) $$F1=2\;\cdot \;\frac{\mathrm{Precision}\cdot \mathrm{Recall}}{\mathrm{Precision}+\mathrm{Recall}}$$

The model is trained on parameters like accuracy, precision, recall, F1-score, and ROC AUC so that it is able to classify the brain tumors robustly and accurately. The long training process makes sure that the model learns not only to be precise but also to be generalizable in nature and hence usable in real-world clinical practice.

## 4 Results

**Figure 4:**
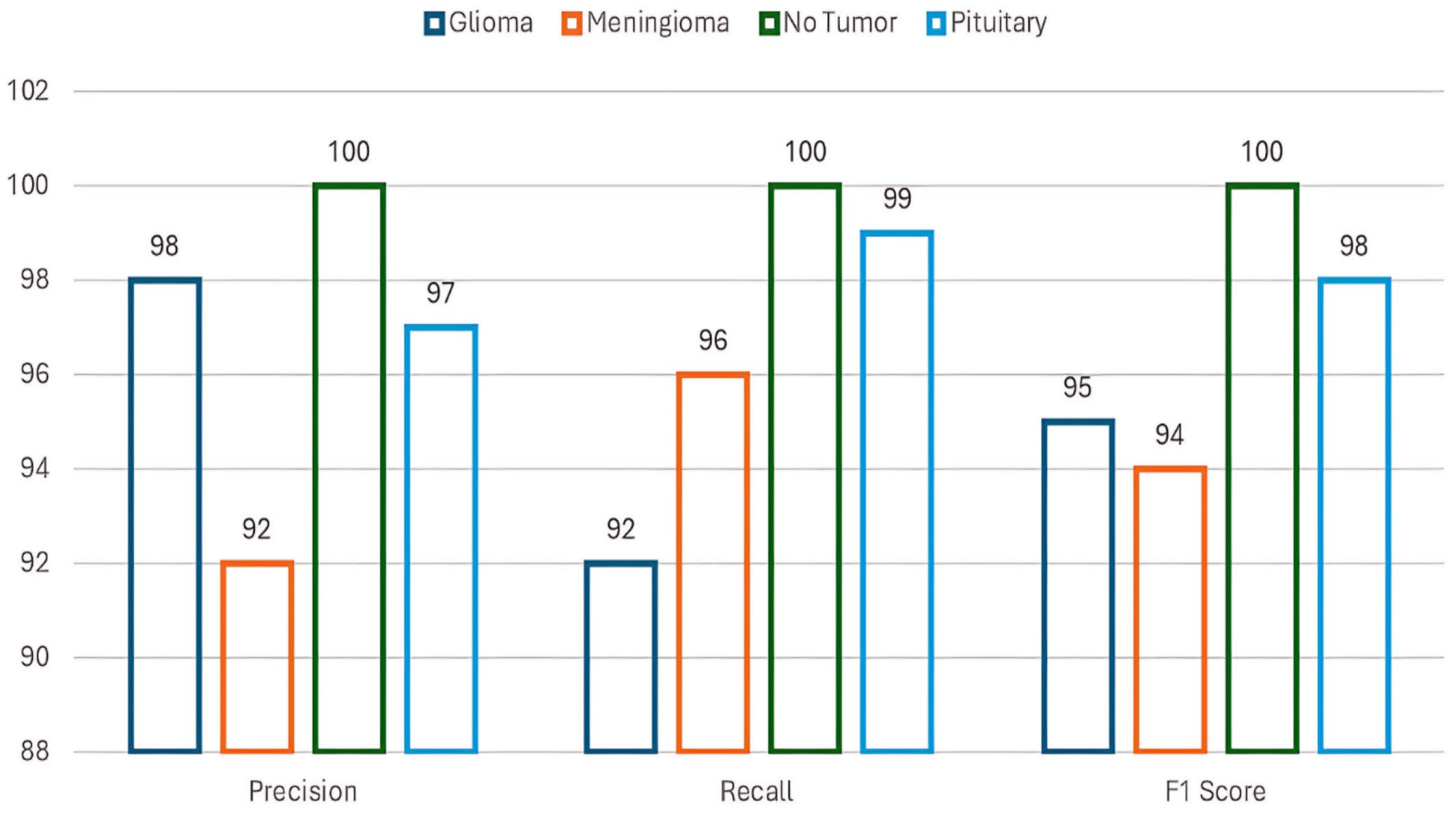
Classification report of the proposed model.

The proposed VGG19-BiLSTM-LightGBM model for brain cancer classification was outstanding in classifying the Brain Tumor MRI Dataset, subjecting it to being able to handle the uncertainty and complexity of the MRI images. The model achieved a training accuracy of 98.69%, validation accuracy of 96.64%, and total test accuracy of 97%, evidence of its ability to generalize to unseen data. Precision, recall, and F1-score metrics also testified to the stability of the model, with its performance being more than 0.92 across all classes. Interestingly, the “No Tumor” and “Pituitary” classes achieved 100% accuracy and recall, while the Glioma and Meningioma classes achieved comparatively lower but still outstanding performance because of their visual similarity. Figure 4 illustrates the categorization report of the suggested model according to all four classes.

**Figure 5:**
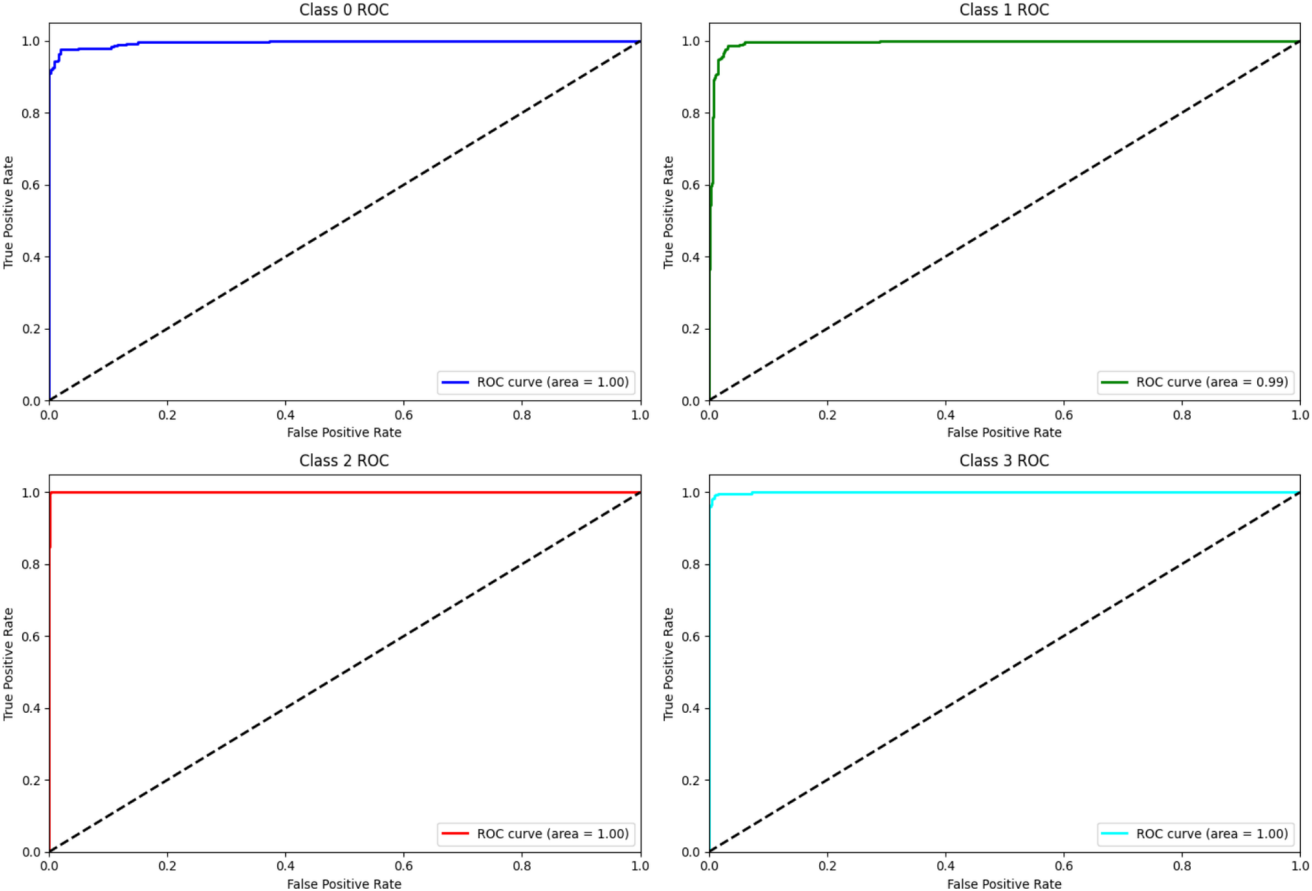
ROC and AUC curve.

The model’s discriminative ability was confirmed by an ROC AUC score of 0.997, indicating its strong capability to distinguish between different tumor types. Figure 5 shows the ROC AUC score of all four classes.

**Figure 6:**
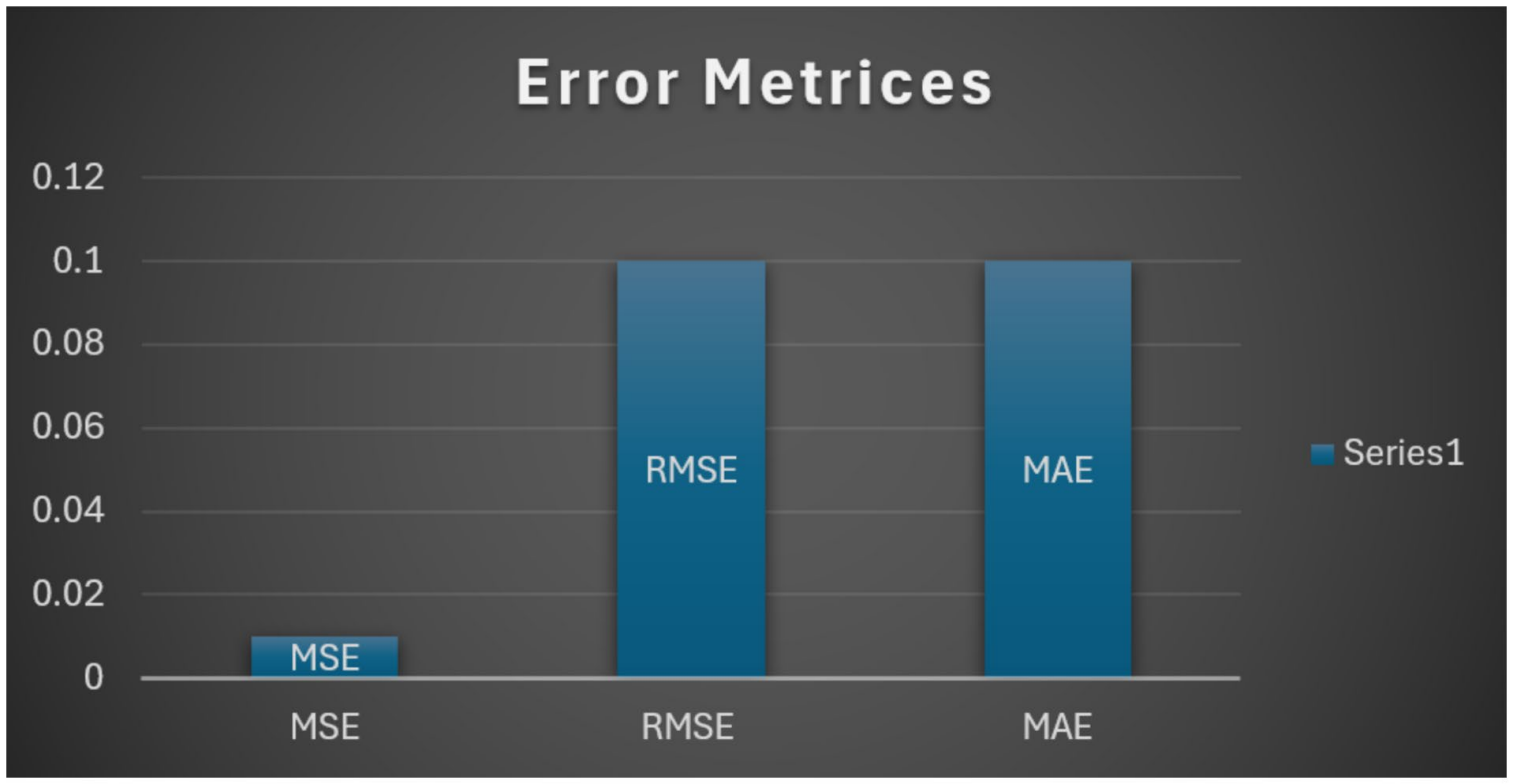
Error metrices.

Error metrics, including Mean Squared Error (MSE = 0.01), Root Mean Squared Error (RMSE = 0.10), and Mean Absolute Error (MAE = 0.10), further underscored the model’s accuracy and reliability. These results demonstrate that the integration of spatial feature extraction (VGG19), sequential modeling (Bidirectional LSTM), and robust classification (LightGBM) provides a powerful framework for brain tumor classification, outperforming traditional single-modal approaches. Figure 6 shows the error metrices of the proposed model.

**Figure 7:**
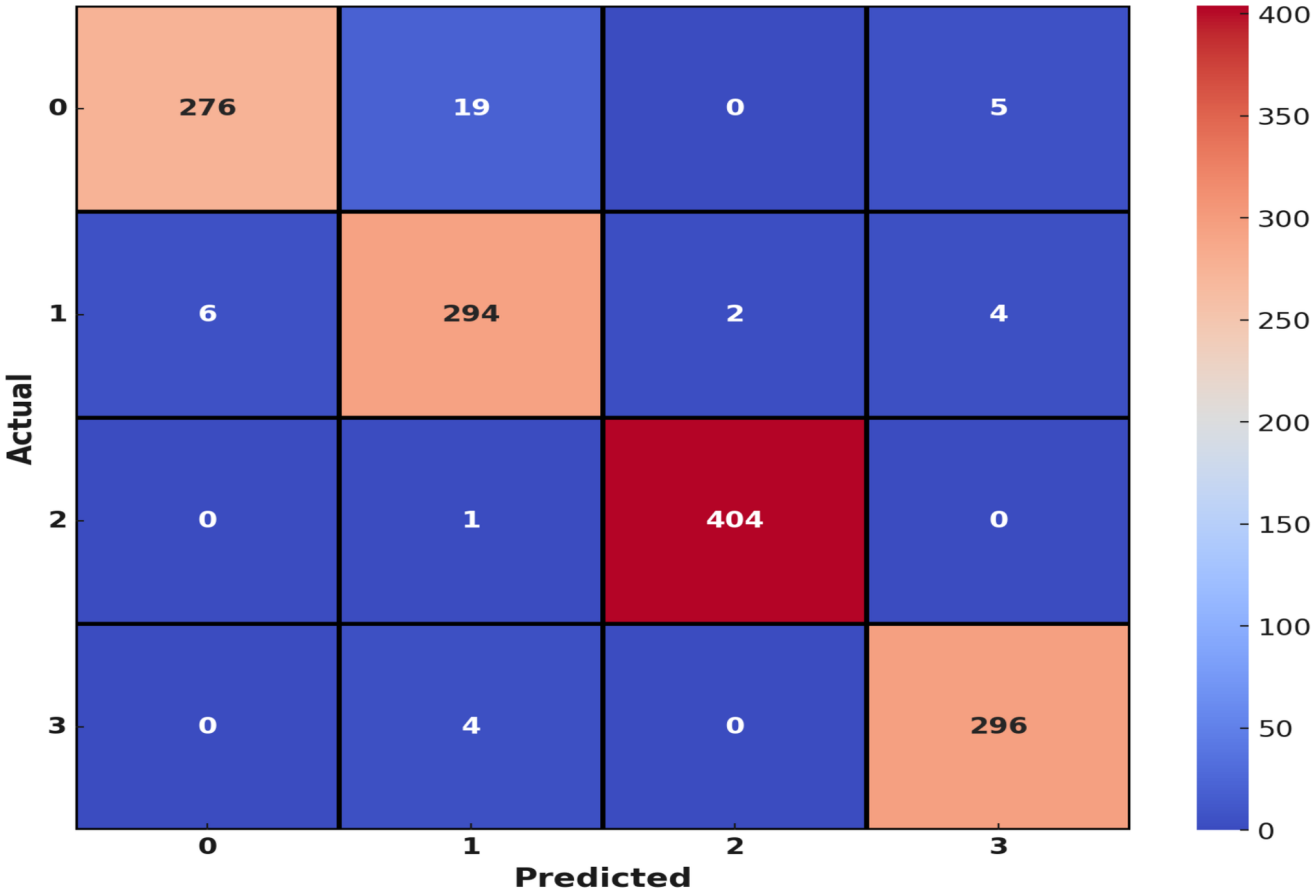
Confusion matrix.

The confusion matrix indicated that the majority of the misclassifications were between the Glioma and Meningioma classes, consistent with the difficulty caused by their visual similarity. The overall misclassification rate was low, and the model performed high accuracy in all classes. The superior performance of the proposed framework compared to baseline procedures, including isolated VGG19 and Random Forest classifiers, supports the advantage of the combination of deep learning and ensemble learning methods. Figure 7 displays the confusion matrix of the utilized dataset.

These findings are important to clinical use as the model has the potential to assist radiologists in more precise and effective diagnosis of brain tumors. But the task can be expanded with other modalities being added, e.g., clinical data or genomic data, to further improve the performance of the model. Table 2 shows the comparison study of many Techniques.

**Table 2:** Comparison study from different techniques.

Study	Techniques	Accuracy
Pan et al. (28)	Convolutional neural networks (CNNs)	96%
Filatov and Yar (29)	EfficientNetB1	89.55%
Ma et al. (30)	CNN	80%
Shilaskar et al. (31)	Extreme gradient boosting (XG Boost)	92.02%
Binish et al. (32)	CBAM	96.70%
Upadhyay et al. (33)	CNN	91%
Ullah et al. (34)	SVM	95.73%
Pandiyaraju et al. (35)	LinkNet architecture	95.84%
Zhu (36)	FT-CNN	96%
Asiri et al. (37) (ML Model)	SVM	95.3%
Stadlbauer et al. (38) (ML Model)	Random forest	0.87%
Proposed model	VGG19-BiLSTM-LightGBM framework	97%

An important development in brain tumor classification is the VGG19-BiLSTM-LightGBM framework, which provides a reliable and expandable solution for medical imaging applications. The VGG19-BiLSTM-LightGBM model achieves excellent accuracy but requires extensive processing resources due to its complex construction. This can result in longer training times and higher costs, which might not be desirable for most clinical scenarios, particularly real-time scenarios. To address this, techniques such as pruning and quantization could be used to reduce model size and speed up inference times without sacrificing accuracy.

## 5 Discussion

In balancing for potential class imbalances in the MRI data sets, a common problem in medical images due to different rates of occurrence of different types of tumors, application of data augmentation techniques and weighted loss function assists in achieving balanced model training and prevents class bias toward majority classes. Scalability of the VGG19-BiLSTM-LightGBM architecture is beyond brain tumor classification. The model’s structure is inherently flexible enough so that it may be utilized to process a wide variety of sickness classes over a large number of imaging modalities. The same structural concepts could reasonably be applied with the goal of classifying chest X-ray abnormalities or skin imaging lesions. This adaptability is primarily attributed to the VGG19 component of the model, which is widely renowned for its capacity to extract informative features from the majority of images, and the very flexible nature of the LSTM and LightGBM components that can be fine-tuned to detect and classify various pathological features with high efficiency. This approach should be applied in low-resource environments. Techniques such as model simplification, quantization, and the use of light-weight neural networks can sufficiently reduce the computational requirements. These parameters are important in maintaining the diagnostic integrity of the model for all categories of tumors. In addition to the computational efficiency and model complexity, there is an inherent trade-off between accuracy and computational requirement. The VGG19, Bidirectional LSTM, and LightGBM together, although computationally expensive, are warranted by the size of accuracy gain and medical diagnostics stability needed. The architecture’s complexity makes it challenging to use in the clinic with real-time requirements.

Existing model implementation into clinical environments may be compromised by latency in processing and loading demands. Future development will center on refining these components to enable real-time analysis, possibly by model reduction or employing more effective processing methods like model quantization and pruning. Future studies will also continue to explore scalability, namely how this system can be adapted or scaled to support different types of tumors or medical imaging tests. This can involve training on larger, more heterogeneous sets of data or modifying the architecture to more effectively encode unique features of individual medical diseases, increasing model flexibility and utility across a broad array of clinical applications.

## 6 Conclusion

The paper offers an important contribution to the brain tumor identification from MRI images using a VGG19-BiLSTM-LightGBM model. The multi-modal approach overcomes complexity and heterogeneity, which are inherently linked to medical imaging data, by using space feature extraction, sequential modeling, and high-performance classification algorithms. Deploying a pre-trained VGG19 model for spatial feature extraction, a Bidirectional LSTM to process sequential information, and LightGBM for efficient and accurate classification, the model improves on diagnostic capability.

With a strong output of 98.69% training accuracy, 96.64% validation accuracy, and 97% test accuracy, it excels over currently available methods such as the VGG19 when isolated and the Random Forest classifier. Such a paradigm, in addition to lowering the chances of error in diagnosis, also aids radiologists in successfully diagnosing brain cancers efficiently and in a timely manner, enhancing patient care. Future upgrades can involve the integration of new data types, e.g., clinical or genetic data, to improve the accuracy as well as the robustness of the model. Additionally, employing explainable AI techniques can enhance the interpretability of the model as a more practical tool for application in clinical contexts. VGG19-BiLSTM-LightGBM is a cost-effective and effective approach to classifying brain tumors and can potentially transform computer-aided diagnosis in radiology.

## Data Availability

The original contributions presented in the study are included in the article/supplementary material, further inquiries can be directed to the corresponding author.
